# Long‐term safety and effectiveness of levodopa‐carbidopa intestinal gel infusion

**DOI:** 10.1002/brb3.758

**Published:** 2017-07-07

**Authors:** Oriol De Fabregues, Joan Dot, Monder Abu‐Suboh, Jorge Hernández‐Vara, Alex Ferré, Odile Romero, Marta Ibarria, José Luis Seoane, Nuria Raguer, Carolina Puiggros, Maria Rosa Gómez, Manuel Quintana, Josep Ramon Armengol, José Alvarez‐Sabín

**Affiliations:** ^1^ Movement Disorders Unit Neurology Department Vall d'Hebron University Hospital Neurodegenerative Diseases Research Group‐Vall d'Hebron Research Institute Autonomous University of Barcelona Barcelona Spain; ^2^ Digestive Endoscopy Department Vall d'Hebron University Hospital Barcelona Spain; ^3^ Sleep Unit Neurophysiology Department Vall d'Hebron University Hospital Barcelona Spain; ^4^ Electromyography Unit Neurophysiology Department Vall d'Hebron University Hospital Barcelona Spain; ^5^ Nutritional Support Department Vall d'Hebron University Hospital Barcelona Spain; ^6^ Pharmacy Department Vall d'Hebron University Hospital Barcelona Spain

**Keywords:** levodopa‐carbidopa intestinal gel infusion, motor fluctuations, nonmotor symptoms, Parkinson, safety

## Abstract

**Introduction:**

Levodopa‐carbidopa intestinal gel (LCIG) infusion has demonstrated to improve motor fluctuations. The aim of this study is to assess the long‐term safety and effectiveness of LCIG infusion in advanced Parkinson's disease (PD) patients with motor fluctuations and its effect in nonmotor symptoms.

**Methods:**

Adverse events (AE) and their management, clinical motor, and nonmotor aspects were assessed up to 10 years. Thirty‐seven patients were treated with LGIC; in three subsets of patients, specific batteries of tests were used to assess cognitive and behavior assessment for 6 months, quality of sleep for 6 months, and quality of life and caregiver burden for 1 year.

**Results:**

There was a high number of AE, but manageable, most of mild and moderate severity. All patients experienced significant improvement in motor fluctuations with a reduction in mean daily *off* time of 4.87 hr after 3 months (*n* = 37) to 6.25 hr after 9 years (*n* = 2). Diskynesias remained stables in 28 patients (75.7%) and improved in 5 patients (13.5%). There was no neuropsychological deterioration, but an improvement in attentional functions, voluntary motor control, and semantic fluency. Quality of sleep did not worsen, and there was an improvement in the subjective parameters, although overnight polysomnography did not change. There was a significant sustained improvement of 37% in PD‐Q39 after 3 months and to 1 year, and a significant reduction in caregiver burden of 10% after 3 months.

**Conclusion:**

LCIG infusion is a safe and efficacious treatment for the control of motor fluctuations, and for improvement or nonworsening of nonmotor aspects, long‐term sustained, and feasible for use in routine care.

## 1 **INTRODUCTION**

A group of treated patients experience motor complications (fluctuations and dyskinesias) when Parkinson's disease (PD) progresses. At this stage of the disease, there are three “second line” device‐aided therapeutic options which may be offered to patients: deep brain stimulation (DBS), subcutaneous infusion of apomorphine (SIApo), and continuous intrajejunal infusion of levodopa‐carbidopa intestinal gel (LCIG) (Martínez‐Martin et al., 2015; Munro Neville, Parsons, Askmark, & Nyholm, 2012).

Optimizing levodopa delivery with LCIG infusion is a treatment option for advanced PD with 10 years in the European market. LCIG infusion has demonstrated to improve motor fluctuations by reducing fluctuations in plasma levodopa levels. The effect of LCIG in other settings has been poorly studied. Some works have demonstrated that treatment with LCIG may improve nonmotor symptoms of PD, may improve cognitive function and behavior (Sanchez‐Castañeda et al., 2009; Zibetti et al., 2013), quality of sleep in these patients (Eggert et al., 2008; Honig et al., 2009), and patient's quality of life (QoL) and caregiver burden (Isacson, Bingefors, Kristiansen, & Nyholm, 2008; Puente et al., 2010; Santos‐García et al., 2012). However, LCIG is a complex and expensive treatment and data on long‐term standard clinical practice therapy complications and their management are scarce.

There is still little known about long‐term follow‐up of LGIC infusion in PD patients. There are only two other 10‐ and 17‐year retrospective studies (Nyholm, Johansson, Lennernäs, & Askmark, 2012; Nyholm et al., 2008), and one 10‐year prospective study (Lim, Schoeman, & Nguyen, 2015). However, the 10‐year prospective study only include follow‐up of the percutaneous endoscopic gastrostomy (PEG) procedure.

The aim of this study is to analyze our long‐term experience in the management of LCIG treatment for PD with motor fluctuations, the safety and effectiveness of this therapy in the control of motor fluctuations, and its effect in other motor and nonmotor symptoms, such as cognitive and sleep disorders, impact in their QoL and caregiver burden, problems found and actions taken to solve them, and reason for treatment discontinuation.

## 2 MATERIALS AND METHODS

### 2.1 Study design and patient selection

This was a long‐term, open‐label, prospective, observational study in 37 patients with advanced PD, responders to levodopa, and with disabling motor fluctuations. All patients included fulfilled the UK Brain Bank criteria (Hughes, Daniel, Kilford, & Lees, 1992) for the diagnosis of idiopathic PD and were experiencing severe motor fluctuations, which were debilitating in daily life, despite receiving optimized conventional oral medications. Patients had been previously treated with oral levodopa combined with entacapone, rasagiline, dopamine agonists, and/or apomorphine injections, 9 of them presented adverse events (AEs) with SIApo and 4 were dismissed for DBS. Patients with atypical parkinsonian features were not included (Wenning et al., 2000).

All PD medication was switched to LCIG (Duodopa^®^ AbbVie) at the start of study treatment. LCIG was initially administered as a continuous duodenal infusion via a nasoduodenal probe using a portable external pump in order to assess individual treatment response and required dose during a test period of 3–10 days (4 days in average). A gastroduodenal catheter was then introduced by PEG for permanent infusion of perfused LCIG. Levodopa‐carbidopa was supplied by the same portable pump via a catheter into the jejunum, with dose delivery individually adjusted to minimize *Off* time periods and dyskinesia during *On* time periods (CADD‐Legacy^®^ Duodopa^®^ PCA‐pump/Smiths Medical ASD/St Paul/MN/USA).

Three open‐label, prospective, observational substudies were carried out in three groups of patients from this population of patients with advanced PD: Nonmotor assessment of cognition and behavior (Substudy 1), nonmotor effects on quality of sleep (Substudy 2), and assessment of health status, QoL, and caregiver burden (Substudy 3).

The study was conducted in compliance with the ethical standards and was approved by the Ethics Committee of the institution (Vall d'Hebron University Hospital), and followed the Spanish Law 15/1999 on Personal Character Data Protection concerning confidentiality of Patient's data. All patients participating in the study signed the corresponding written consent form.

### 2.2 Clinical evaluation/assessments

**Figure 1:**
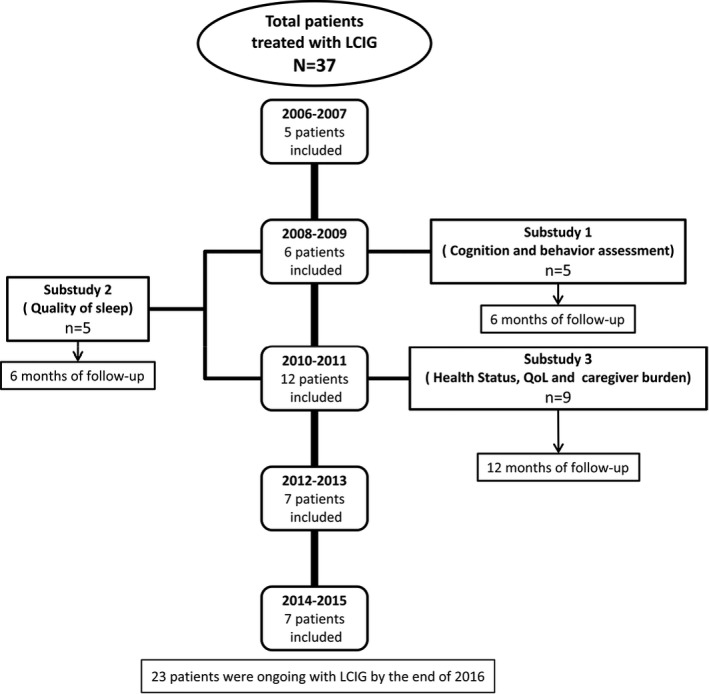
Flow chart showing inclusion of patients along time

The following parameters were analyzed prior to LCIG treatment (at baseline), at months one, three, six, and twelve, and every year afterward over a 10‐year period (from May 2006 to May 2016) (see Flow chart on Figure 1):

- Safety:
  o AEs related to PEG procedures and gastrostomy, infusion device, and treatment.
  o Severity of AEs evaluated as mild (event well tolerated by patient, causing minimal discomfort, and not interfering with daily activities), moderate (the event causes sufficient discomfort to interfere with daily activities), and serious (the event impedes daily activities, results in death, is life threatening, requires inpatient hospitalization or causes prolongation of existing hospitalization, results in persistent or significant disability/incapacity, is a congenital anomaly/birth defect, or requires intervention to prevent permanent impairment or damage).
  o Actions taken to solve them, and reasons for treatment discontinuation and withdrawal.
- Effectiveness:
  o Motor fluctuations: *Off* time in hours recorded in Parkinson's Disease Diary©.
  o Dyskinesia and other motor clinical aspects: evaluated with the Unified Parkinson's Disease Rating Scale (UPDRS) part IV, UPDRS part II in *On* and *Off*, UPDRS part III in *On* and *Off*, Hoehn and Yahr (H&Y) stage in *On* and *Off*, and Schwab and England (S&E) scale in *On*.
  o Nonmotor clinical aspects: cognitive function through Mini Mental State Examination (MMSE), and UPDRS part I, and relevant neuropsychiatric disorders.

The parameters analyzed in the three prospective substudies carried out in three subsets of this population are described below:

*Substudy 1—Cognition and behavior assessment*: Subgroup of patients consecutively included between December 2008 and January 2009, evaluated with a specific neuropsychological battery for assessment of cognition and behavior disorders prior to treatment (at baseline) and after 6 months of LCIG, by the same neuropsychologist at the same environmental conditions and in patients in phase *On*. The cognitive examination included: tests that assessed cognitive areas affected in PD according to the literature, psychometric tests with well‐known parameters, tests that can be used in different types of populations (neurologic and psychiatric disorders, screening, etc.), and tests suitable for a population with low educational and cultural level.

- Attentional function: Forward Digit Span test of Weschler Adult Intelligence Scale‐Third Edition (WAIS‐III); Audio‐verbal attentional capacity; and Stroop Color‐Word test.
- Executive functions: Backward Digit Span of WAIS‐III; Audio‐verbal working memory; Stroop‐word and Stroop‐color subtests; Response inhibition capacity; Controlled Oral Word‐Association Test (FAS) of phonemic verbal fluency; and Category Naming Test (Animals) of semantic verbal fluency.
- Visual‐constructional visuospatial and visuoperceptual functions: Clock Drawing Test*—*(order and copy)*—*visual‐constructional; Reading clocks*—*simple visuospatial ability; and Luria test of overlapping figures*—*visual perceptive skills function.
- Memory and learning: Rey Auditory‐Verbal Learning Test (RAVLT)*—*Short‐ and long‐term audio‐verbal memory and recognition.
- Language: Boston Naming Test (BNT)*—*Title by visual comparison.
- Motor functions: Luria motor sequences*—*Voluntary motor control, Motor coordination.
- Mood: Beck depression inventory (BDI)
- Behavior: Neuropsychiatric Inventory (NPI)*—*Exploration of psychological and behavioral symptoms.

*Substudy 2—Quality of sleep*: Subgroup of patients consecutively included between January 2009 and June 2010, evaluated with Epworth scale, fatigue scale, Pittsburg quality of sleep questionnaire, BDI, and Hamilton anxiety scale, administered prior to treatment (at baseline) and 6 months after treatment. In addition, an overnight polysomnography (PSG) study was carried out at these timings.

*Substudy 3—Health status, QoL, and caregiver burden*: Subgroup of patients consecutively included between June 2010 and June 2011, evaluated for up to 12 months, with the Spanish version of the 39‐item quality‐of‐life questionnaire in PD (PDQ‐39, 0–156), health status questionnaires (EQ‐5D, range 5–15; and EQ‐VAS range 0–100), global clinical impression scale (CGI, range 1–7), and caregiver burden questionnaire or Zarit Burden index (ZBI, range 0–100). Assessments were done prior to treatment (at baseline), 1 week, 3 months, 6 months, and 12 months after treatment.

### 2.3 Statistical analysis

Statistical analysis was carried out using the SPSS statistical package v17.0 for Windows. A *p* value <.05 was considered statistically significant.

Variables were expressed as frequency (percentages) in categorical variables and mean ± standard deviation (SD) or median (range) in numerical variables. Normal distributions of continuous variables were assessed by the Kolmogorov–Smirnov test and Q–Q plot.

Paired‐samples *t*‐test and Wilcoxon signed‐rank test were used for group comparisons of continuous variables. McNemar's and McNemar–Bowker tests were used to analyze changes in categorical variables.

## 3 RESULTS

### 3.1 Baseline characteristics and LCIG treatment administration

Thirty‐seven patients were included (22 males, 15 females) to treatment with LCIG. The mean age was 68.2 ± 6.8 years (57–80) and the mean duration of the disease was of 13.5 ± 5.6 years (5–26).

Prior to LCIG treatment, patients presented a daily mean *Off* time of 6.0 ± 1.4 hr with *On* H&Y of 16.2% in stage 2, 67.5% in stage 2.5, and 16.2% in stage 3; and with *Off* H&Y of 35.1% in stage 3, 51.4% in stage 4, and 13.5% in stage 5. Total UPDRS score was 43.2 ± 15.7 in *On* stage and 73.4 ± 21.8 in *Off* stage. Patients presented diskynesias from 1% to 25% of the day in 14 patients (37.8%), from 26‐50% in 19 patients (51.4%), and from 51% to 75% in four patients (10.8%); which were nondisabling in four patients (10.8%), mildly in 19 patients (51%), moderately in 12 patients (32.4%), and severely disabling in two patients (5.4%). The median MMSE score was 28 (20–30). There were neuropsychiatric nonmotor symptoms in 20 patients (54.1%), nine patients with cognitive impairment (24.3%), nine with confusion (24.3%), 11 with visual hallucinations (29.7%), four with delusions and psychotic disorders (10.8%), and eight with impulsive and compulsive behaviors (21.6%).

All patients received LCIG treatment for an average of 43.6 ± 31.5 months (1–120 months). From the 37 patients receiving LCIG treatment, 1 (2,7%) arrived to 10‐year control, 2 (5.4%) to 9‐year control (108 months), 13 (35.1%) to 5‐year control (60 months), 23 (62.2%) to 2‐year control (24 months), and 30 (81.1%) to 1‐year control (12 months).

### 3.2 Safety assessment

Patients presented a high number of AEs, mainly related to the device and the infusion system, but also to the PEG procedure and the gastrostomy as well as to the treatment, with a similar profile as described for oral levodopa (Table 1).

**Table 1:** Adverse events (AEs) and actions taken

AEs related with	Type	Number (%) of patients	Severity	Action taken
PEG procedures Gastrostomy	Abdominal pain, nausea, and vomiting	12 (32.4%)	Mild–Moderate	Analgesic
	Local peritonitis post‐PEG	5 (13.5%)	Mild–Moderate	Systemic antibiotic
	Pneumoperitoneum post‐PEG	3 (8.1%)	Moderate	Diet
	Granuloma	14 (37.8%)	Mild	Topical treatment
	Stoma dermatitis	12 (32.4%)	11 Moderate 1 Serious	Topical treatment
	Stoma leakage	2 (5.4%)	Moderate	Topical treatment
	Stoma infection	7 (18.9%)	4 Moderate 3 Serious	Systemic antibiotic PEG removal
Infusion device	PEG replacement	34 (91.2%)	Moderate	Endoscopy and replacement
	Connection breakage or failure	10 (27.0%)	Mild	Replacement
	External tube breakage	2 (5.4%)	Mild	Replacement
	PEG removal	4 (10.8%)	Serious	Maintaining gastrostomy Endoscopy and repositioning or replacement
	Exterior output of intestinal tube	11 (29.7%)	Moderate	Endoscopy and repositioning or replacement
	Transitory obstruction of intestinal tube	13 (35.1%)	Mild	Tube washing, prokinetic treatment
	Permanent obstruction of intestinal tube	13 (35.1%)	Moderate	Tube washing, Endoscopy, and repositioning or replacement
	Internal migration of intestinal tube	5 (13.5%)	Moderate	Endoscopy and repositioning or replacement
	Migration of intestinal tube head to stomach	2 (5.4%)	Moderate	Prokinetic treatment Endoscopy and replacement
	PEG hooked—foreign body reaction	3 (8.1%)	Serious	2 Removal and new gastrostomy 1 LCIG withdrawal
	Ulceration	2 (5.4%)	Moderate	Treatment with proton pump inhibitors
	Pump malfunctioning	9 (24.3%)	Moderate	Replacement
Pharmacological	Leg pain	15 (40.5%)	11 Mild 3 Moderate 1 Serious	Dose adjustment
	Polyneuropathy (PNP)	13 (35.1%)	12 Mild 1 Serious	Dose adjustment, vitamin supplement, symptomatic treatment
	Freezing, dystonia of leg in *On*	7 (18.9%)	3 Mild 2 Moderate 2 Serious	Dose adjustment
	Biphasic dyskinesias	6 (16.2%)	1 Mild 2 Moderate 3 Serious	Two 24‐h LCIG dose adjustment 1 LCIG withdrawal
	Confusion	11 (29.7%)	3 Mild 2 Moderate	Dose adjustment
	Hallucinations, psychosis	13 (35.1%)	7 Mild 6 Moderate	Dose adjustment, neuroleptic treatment, ACE inhibitors
	Impulsive and compulsive behavior	8 (21.6%)	5 Mild 2 Moderate	Dose adjustment, neuroleptic treatment
	Significant hypotension	5 (13.5%)	1 Mild 4 Moderate	Dose adjustment, coffee, salt, fludrocortisone
	Vitamin B12 deficit	12 (32.4%)	NA	Vitamin supplement
	Vitamin B6 deficit	5 (13.5%)	NA	Vitamin supplement
	Homocysteine excess	11 (29.7%)	NA	Vitamin supplement
	Weight loss	9 (24.3%)	1 Mild 5 Moderate 3 Serious	Diet

Results in frequency (percentage).

NA, not available, intensity not collected; LCIG, levodopa‐carbidopa intestinal gel; PEG, percutaneous endoscopic gastrostomy.

Most of the AEs were of mild and moderate severity, and serious in a minor degree. Serious complications related to PEG procedures and gastrostomy were 1 stoma dermatitis and 3 stoma infection; 4 PEG removal and 3 PEG hooked related to infusion device; and 1 leg pain, 1 polyneuropathy (PNP), 2 freezing in *On*, 3 dyskinesia, and 3 weight loss related to LCIG treatment.

Although treatment was temporary discontinued as a consequence of some of these AEs, they were manageable and actions taken allowed continuing treatment in most cases (Table 1). Treatment was permanently discontinued in 14 patients (37.8%): seven patients (18.9%) who died due to multiple comorbidity with other diseases; three patients (8.1%) with disease progression with dementia and worsening of *On* stage and decrease in the change between *On* and *Off* stage; and four patients (10,8%) with AEs such as intolerance to the administration system in two patients (5.4%), serious stoma infection in one patient (2.7%), and worsening of dyskinesia in one patient (2.7%). Only one patient received rescue with SIApo and one with DBS.

### 3.3 Effectiveness assessment

**Figure 2:**
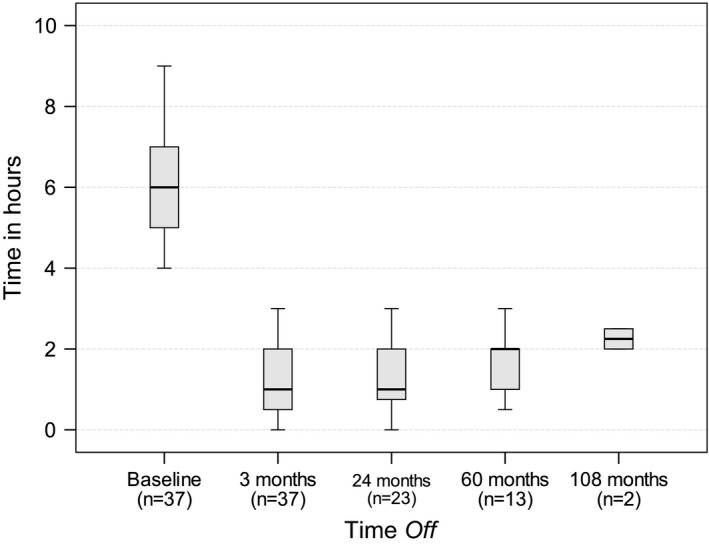
Time *Off* from baseline to 108 months

After LCIG treatment, all patients showed a significant and sustained motor improvement in motor fluctuations (Figure 2), with a reduction in daily mean *Off* time of 4.9 ± 1.1 hr after 3 months (*p *< .001) in 37 patients, 4.9 ± 1.1 after 2 years (*p *< .001) in 23 patients, 5.0 ± 1.1 after 5 years (*p *< .001) in 13 patients, and 6.3 ± 0.4 after 9 years in two patients (*p* = .025).

Regarding dyskinesia, none of the patients presented a worsening in the percentage of the waking time with dyskinesia, which was reduced in 6 patients (16.1%) and remained stable in the remaining 31 patients (83.8%). The changes in severity were not statistically significant (*p* = .176), severity of dyskinesias improved in 5 (13.5%), remained stable in 28 (75.7%), and worsened in 4 (10.8%) patients.

H&Y motor stages significantly improved after 3 months of treatment. *On* stages improved in 10 (27%) patients from stage 2.5 to stage 2 (*p *< .002), the remaining 27 (73%) remained stable, none was worsened. *Off* stages improved in 17 (45.9%) patients from stage 4 to stage 3 (*p *< .001), and the remaining 20 (54.1%) remained stable, none worsened at midterm.

The median S&E score of 50 pretreatment, where patients had major dependence and need partial help, significantly improved to 80 after 3 months of treatment, being patients independent in most of the daily activities (*p *< .001).

Motor symptoms evaluated with UPDRS part III remained stable with a slight improvement only significant for *Off* stage at midterm. The mean score in *On* stage changed from 22.2 ± 8.4 pretreatment to 21.1 ± 8.8 after 3 months of treatment (*p* = .080), and the mean score in *Off* stage changed from 40.9 ± 13.2 pretreatment to 39.0 ± 12.0 after 3 months of treatment (*p* = .047).

Nonmotor cognitive function and behavior evaluated with UPDRS part I improved from a mean score of 3.2 ± 2.4 pretreatment to 2.5 ± 1.7 after 3 months of treatment (*p *< .001). However, no significant differences were found in cognitive function evaluated by MMSE, with a median score of 28 pretreatment and 29 after 3 months of treatment (*p* = .655). Neuropsychiatric disorders (mental confusion, visual hallucinations, delirium and psychotic disorders, impulse control disorder with compulsive and impulsive behavior, pathological gambling, compulsive buying, punding, and dopaminergic dysregulation syndrome) persisted but did not worsen in general and impulse and compulsive behavior improved, without any new case of this complication during the follow‐up.

### 3.4 Substudy 1*—*Cognition and behavior assessment

This substudy included five patients (three males, two females) with a mean age of 69.6 (60–73) years and a mean disease duration of 14.4 (8–22) years.

No statistical significant differences were found between baseline scores and after 6 months of treatment in any of the neuropsychological tests, despite most scores tend to be maintained or improved in some tests. After LCIG there was an improvement of 5 points in verbal memory, short‐ and long‐term attentional functions, voluntary motor control, phonetic verbal fluency, and naming (Table 2). Regarding behavior, no differences between assessments were found.

**Table 2:** Cognitive function*—*neuropsychological assessment (Substudy 1)

Patients (*n* = 5)	Pretreatment (Baseline)	Posttreatment (6 months)	*p* value
Phonemic fluency (FAS)	18.2 ± 17.1	19.6 ± 15.8	.465
Semantic fluency (Animals)	12.8 ± 4.6	13.2 ± 5.6	.655
Boston Naming Test 30	21.8 ± 6.1	22.4 ± 4.4	.854
WAIS‐III Digits Forward	6.4 ± 2.5	6.8 ± 1.6	.581
WAIS‐III Digits Backward	3 ± 1.7	3.6 ± 1.5	.276
RAVLT A1	2.6 ± 1.1	3.2 ± 0.8	.480
RAVLT A2	3.8 ± 2.2	4.6 ± 1.5	.180
RAVLT A3	5.0 ± 1.7	5.4 ± 1.7	.705
RAVLT A4	5.8 ± 2.5	7.2 ± 3.2	.465
RAVLT A5	6.6 ± 2.3	8.0 ± 3.7	.414
RAVLT A7	3.2 ± 3.0	5.6 ± 4.2	.066
Recognition RAVLT	10.0 ± 6.0	11.8 ± 2.6	.854
Motor sequences	14.6 ± 8.4	19.4 ± 13.7	.276
Reciprocal coordination	16.6 ± 11.7	17.2 ± 10.0	.715
The clock test–reading	12.6 ± 3.9	12.8 ± 2.1	1.000
Luria Test of Overlapping Figures	13.2 ± 5.94	12.8 ± 1.3	1.000
Stroop‐word	58.6 ± 25.7	69.5 ± 38.9	1.000
Stroop‐color	45.2 ± 24.8	36.0 ± 26.2	.461
Stroop‐word color	18.5 ± 9.8	24.0 ± 16.5	.465
Stroop‐word color errors	0.7 ± 0.9	3.0 ± 3.6	.357
The clock test–drawing	5.0 ± 2.1	4.2 ± 2.6	.102

Results in mean ± standard deviation.

FAS, Verbal Fluency Test with words that start in F‐A‐S; WAIS, Weschler Adult Intelligence Scale; RAVLT, Rey's Auditory‐verbal learning test; A1–A5, Assay 1–5, A7, differed audio‐verbal memory.

### 3.5 Substudy 2*—*Quality of sleep

This substudy included five patients (one male, four females) with a mean age of 69 (60–76) years and a time of evolution of PD of 14 (8–15) years. Before starting treatment with LCIG, the quality of sleep was bad either in objective and subjective parameters (Table 3); with a mean score for the Pittsburg scale of 10.2 (Normal values <5 and Severe values >14), and a mean score for periodic leg movement (PLM) of 15.0 (Normal values <15), and 3 of the 5 patients presented a REM phase without atony.

**Table 3:** Polysomnography parameters*—*quality of sleep (Substudy 2)

Patients (*n* = 5)	Pretreatment (Baseline)	Posttreatment (6 months)	*p* value
*Objective parameters*			
Efficiency	66.2 ± 9.3	55.2 ± 18.3	.225
Waking up during sleep	124.3 ± 82.0	100.8 ± 60.9	.144
Sleep latency	33.6 ± 44.0	89.9 ± 112.1	.686
REM latency	164.1 ± 71.4	150.2 ± 78.4	.715
REM%	13.6 ± 8.5	11.6 ± 7.3	.144
N1%	18.0 ± 10.7	25.2 ± 16.2	.686
N2%	54.3 ± 8.5	47.6 ± 5.0	.043^a^
N3%	14.0 ± 8.4	15.7 ± 13.1	.686
Snoring (n/h)	184 ± 275.2	285 ± 295.9	.109
AHI	3.0 ± 3.2	4.6 ± 2.9	.593
Microawakenings	12.9 ± 5.6	10.0 ± 3.2	.225
PLM	15.0 ± 11.0	10.8 ± 12.0	.345
Baseline oximetry	94.4 ± 2.3	95.2 ± 2.1	.414
Mean oximetry	93.6 ± 2.4	94.2 ± 2.6	.461
Minimum oximetry	91.8 ± 2.5	89.0 ± 5.0	.194
CT90	0.6 ± 1.3	0.6 ± 0.6	.109
*Subjective parameters*			
Epworth	5.6 ± 3.6	2.8 ± 1.7	.131
Subjective efficiency	66.7 ± 24.3	70.6 ± 23.2	.273
Pittsburg	10.2 ± 6.9	8.4 ± 6.0	.461
Fatigue scale	39.4 ± 15.2	37.4 ± 17.9	.465
Beck depression scale	9.4 ± 7.6	11.2 ± 7.6	.786
Hamilton anxiety scale	20.40 ± 12.6	19.0 ± 13.7	.485

Results in mean ± standard deviation.

*N,* Sleep stages; AHI, Apnea Hypopnea Index; PLM, periodic leg movement; CT90, oxygen saturation below 90%.

a *p *< .05 indicate significant differences compared to baseline.

PSG showed a low efficiency of sleep in these PD patients. No significant differences were found in the macrostructure of sleep, respiratory events, and PLMs after 6 months of treatment (Table 3).

The subjective questionnaire on somnolence showed that patients with somnolence improved after treatment, although not significantly. There was an improvement of 5 points in the Epworth scale without deterioration of the subjective parameters of quality of sleep, depression, fatigue, anxiety, or objective parameters of overnight PSG.

### 3.6 Substudy 3*—*QoL and caregiver burden

This substudy included nine patients (eight males, one female) with a mean age of 69.6 (57–78) years and a mean disease duration of 14.4 (8–23) years.

There was a significant global clinical improvement, improvement of QoL and health status, and lower healthcare burden after treatment with LCIG (Table 4). There was a sustained improvement in PD‐Q39 questionnaire of 37% at 3, 6, and 12 months with a mean decrease of 21 points (*p *< .05). EQ‐5D questionnaire significantly improved at 1 week and at 3 and 12 months with a mean decrease of 2 points (improvement of 20%, p* *< .05). EQ‐VAS scale was significantly better at 1 week and at 3 months with an increase of 14 points (improvement of 25%, *p *< .05). The caregiver burden evaluated by ZBI significantly improved a 20% at 3 months with a mean decrease of 8.7 points (*p* = .042), being the improvement lower and nonsignificant after 1 year (10% improvement with a mean decrease of 3.2 points).

**Table 4:** QoL, health status, and caregiver burden scales (Substudy 3)

Patients (*n* = 9)	Pretreatment	Posttreatment							
	Baseline	1 week	*p* value	3 months	*p* value	6 months	*p* value	1 year	*p* value
PDQ‐39	56.9 ± 11.4	41.9 ± 21.5	.097	35.7 ± 18.6	.021^a^	35.5 ± 19.1	.021^a^	35.5 ± 18.8	.018^a^
EQ‐5D	9.3 ± 1.7	7.9 ± 2.6	.041^a^	7.5 ± 2.1	.026^a^	8.2 ± 2.5	.140	7.5 ± 1.9	.042^a^
EQ‐VAS	54.9 ± 11.7	71.7 ± 6.9	.017^a^	68.7 ± 7.7	.027^a^	64.3 ± 13.6	.249	66.2 ± 9.9	.068
ZBI	30.9 ± 17.7	26.0 ± 17.8	.173	22.2 ± 10.8	.042^a^	27.5 ± 16.2	.074	27.7 ± 15.5	.058

Results in mean ± standard deviation.

PDQ‐39, quality‐of‐life questionnaire in PD—39 items; EQ‐5D, European Quality of life—5 dimensions; EQ‐VAS, European quality‐of‐life Visual Analogue Scale; ZBI, Zarit Burden Index.

a *p *< .05 indicate significant differences compared to baseline.

## 4 DISCUSSION

This is the first and longest follow‐up prospective study carried out in Spain on long‐term effects of LGIC infusion in PD patients.

Our safety profile was consistent with previous studies (Cáceres‐Redondo et al., 2014; Nyholm et al., 2008; Zibetti et al., 2014). The most frequent issues in our study were related to the infusion device and mainly of mild intensity, although we also found serious complications such as PEG removal in four patients and PEG hooked in three patients. Most of these complications were preventable with annual/biennial PEG replacement. Complications related to PEG procedures and gastrostomy were also frequent and generally mild, although there also were serious issues such as stoma dermatitis in one patient and stoma infection in three patients. All complications were solved with topical/systemic treatment, and only in one patient lead to treatment permanent discontinuation (this same patient presented PEG hooked, and has a deficient hygiene and progressive dementia).

With regard to AEs related to treatment, there were 13 patients with PNP, four cases already present prior to treatment, four cases of small fiber PNP, four cases of axonal subacute PNP, and one case of serious acute axonal PNP. Almost all cases evolved to stabilization or improvement except the acute PNP case who was stabilized but with neurologic sequelae. Presence of PNP was already described with long‐term treatment at high doses of oral levodopa (Puente et al., 2010), present either as Guillain‐Barré syndrome (Antonini et al., 2007), or as axonal PNP in the context of vitamin B12 deficit or other group B vitamins (Manca et al., 2009; Santos‐García et al., 2010, 2012). In our study, most of the patients improved with vitamin B12 and B6 supplements. Weight loss was another relevant complication in our patients, treated with diet or addition of the corresponding supplements, being therefore important to verify the nutritional status of the patients. Worsening of biphasic dyskinesias was particularly severe in three patients, solved in two cases with dose adjustment, but in one case LCIG treatment was removed.

Despite the complexity of the treatment and the high number of AEs occurred, as most AEs were manageable and with the good effectiveness results found, LGIC treatment may be maintained over a period of 10 years so far. In fact, in our study LGIC treatment has been used as rescue treatment for nine patients previously receiving SIApo and four previously receiving DBS, while within patients receiving LGIC only one received rescue treatment with SIApo and one with DBS.

Several studies have shown the effectiveness of LCIG therapy in motor and nonmotor fluctuations in the standard clinical practice in PD (Buongiorno et al., 2015; Eggert et al., 2008; Fernandez et al., 2015). In our study, a significant reduction in mean daily *Off* time of 4.8 hr was found in all patients after LCIG treatment, which is slightly higher than the reduction in mean daily *Off* time of 4.4 hr found in a 12‐month study (Fernandez et al., 2015), and of 4 hr in a 12‐week follow‐up study (Olanow et al., 2014). Our slightly higher results are probably because the standard clinical practice allows greater agility and versatility in the treatment.

Improvement of dyskinesias with long‐term treatment with LCIG has been confirmed in several studies up to 7 years with significant reductions in *On* time during waking time without incapacitating dyskinesias (Antonini et al., 2013; Santos‐García et al., 2010), and global improvement of dyskinesias (Cáceres‐Redondo et al., 2014; Olanow et al., 2014; Timpka et al., 2016; Zibetti et al., 2014), while other studies did not confirm this results (Nyholm et al., 2008) or reported clinical worsening of patients in a short series of patients (Raudino et al., 2009). In our study the percentage of waking time with dyskinesias was reduced in six patients and maintained in the remaining 31 patients, and severity of dyskinesias improved in five patients, remaining stable in 28, but worsening in four being a reason for withdrawal in one of them.

Furthermore, our study found a significant improvement in motor stages measured with H&Y and motor functions independence and the ability to perform daily activities measured by S&E scale at midterm. Our study is not designed to compare the progressive motor deterioration of natural disease evolution, and therefore we compare with the same population prior to treatment and at midterm (3 months). Although most studies report a global improvement in daily activities and motor complications subscales (Olanow et al., 2014; Pålhagen et al., 2012; Puente et al., 2010; Slevin et al., 2015), other studies report no motor symptom improvement (Antonini et al., 2007; Cáceres‐Redondo et al., 2014; Fasano, Ricciardi, Lena, Bentivoglio, & Modugno, 2012; Sensi et al., 2014) and even worsening at long term up to 3‐year follow‐up (Zibetti et al., 2013).

In line with precedent studies (Eggert et al., 2008; Honig et al., 2009) we also found significant improvement of nonmotor symptoms, particularly those concerning cognitive and behavior function assessed with UPDRS part I. The Non‐Motor Symptoms Assessment Scale for Parkinson's disease was not used because it was not yet validated before the start of our prospective study.

In our experience, nonmotor neuropsychiatric disorders, which are frequent in this population, persisted with treatment but did not worsen in general. Impulse control disorders improved and there was not a single new case occurring. A similar experience was recently found in a 6‐month prospective study (Catalán et al., 2013) and in a 3‐year prospective study (Todorova, Samuel, Brown, & Chaudhuri, 2015). Assessment of mental status with MMSE did not find any worsening with treatment at midterm, as in other studies (Fasano et al., 2012; Pickut, van der Linden, Dethy, Van De Maele, & Zegers de Beyl, 2014).

Regarding the substudy of cognition and behavior assessment, after LCIG treatment, patients with advanced PD had no impairment of cognitive function and there were some improvements in attention, semantic fluency, and voluntary motor control. Although statistical power was low due to the small sample size, there was no evidence that patients undergoing this treatment get worse cognitively, being even an improving trend in some of the scores. LCIG can be considered a complex treatment strategy in advanced PD which do not deteriorate and even can offer some improvement in neuropsychological function.

Several prospective studies reported significant improvements in sleep disorders (Eggert et al., 2008; Fasano et al., 2012; Honig et al., 2009), suggesting that LCIG can improve PD symptoms during night, sleep fragmentation, and dystonic pain. A recent retrospective analysis of 185 patients receiving LCIG therapy found an improvement in >50% of patients in several nonmotor symptoms including sleep disorders (Valldeoriola et al., 2016). Our quality of sleep substudy, although with small sample size, confirmed the presence of bad quality of sleep, mild depression, and anxiety in our patients at baseline. LCIG did not deteriorate the objective parameters of overnight PSG or the subjective parameters of sleep quality, depression, fatigue, and anxiety, and discretely improved Epworh in those patients with Epworth within a pathological range.

The effectiveness of the treatment was also evidenced with a significant global clinical improvement, a significant improvement in their QoL and health status, and lower caregiver burden. LCIG significantly improved a 26% the QoL assessed by PDQ‐39. We obtained similar results than other studies with follow‐up periods up to 2 years (Antonini et al., 2008, 2013; Fasano et al., 2012; Fernandez et al., 2015; Santos‐García et al., 2012; Slevin et al., 2015). Neurodegenerative diseases, such as PD, have a considerable social burden, particularly for the caregiver. In our study LCIG treatment reduced in a 20% the caregiver burden at midterm (3 months) and a 10% at long term (1 year). Although the statistical potency was low due to the small sample size, there were no evidences that patients undergoing this treatment, despite the limitations and complications, had worse QoL or worse caregiver burden. Results from other studies found a tendency toward an improvement in caregiver burden with LCIG treatment (Cáceres‐Redondo et al., 2014; Fasano et al., 2012; Olanow et al., 2014; Sensi et al., 2014; Slevin et al., 2015).

The safety and effectiveness results are particularly important due to the long‐term prospective follow‐up, leading to a better knowledge of LCIG therapy in all aspects, both motor and nonmotor, and on its complications. This is the major strength of this study. The study provides valuable information obtained in the standard clinical practice conditions, and will help to optimize treatment for patients currently receiving or who will receive in the future LCIG treatment. One important limitation in this study is the small sample size in some of the substudies, which determines a low statistical power in the comparisons. Therefore, the results found should be interpreted with caution.

LCIG may be considered a complex treatment strategy for advanced PD with motor fluctuations where drug combinations currently available did not provide satisfactory results. Considering the high cost of LCIG treatment, the potential serious AEs, and their complexity, the most likely candidates for this treatment should be identified and a multidisciplinary specialized and protocolized management is recommended together with the patient cooperation and his/her caregiver or assistant support.

Our longest follow‐up prospective study confirms that LGIC treatment is an efficacious treatment for the control of motor fluctuations, and for improvement or nonworsening of other motor and nonmotor aspects of PD, being well tolerated and safe, long‐term sustained, and feasible for use in the standard clinical practice.

## CONFLICT OF INTEREST

Oriol De Fabregues and Jorge Hernández‐Vara received honoraria from AbbVie, Zambon, and Italfarmaco. Joan Dot, Monder Abu‐Suboh, Alex Ferré, Odile Romero, Maria Ibarria, José Luis Seoane, Nuria Raguer, Carolina Puiggros, Maria Rosa Gómez, Manel Quintana, Josep Ramon Armengol, and José Alvarez‐Sabín reported no financial interests or potential conflicts of interest.
